# Awareness and Utilization of Maternal and Child Health Services Among Women of Reproductive Age in Osogbo, Nigeria

**DOI:** 10.7759/cureus.90850

**Published:** 2025-08-24

**Authors:** Adeniyi Fasanu, Sunday C Adeyemo, Oluwafunmilayo A Fasanu, Daniel A Adekanle, Adenike I Oluwole, Kehinde Awodele, Emmanuel O Folami, Rasaq A Akindele, Eniola D Olabode

**Affiliations:** 1 Obstetrics and Gynecology, Osun State University, Osogbo, NGA; 2 Public Health Research, University of Wolverhampton, Wolverhampton, GBR; 3 Health and Biomedical Sciences, Institut Superieur de Sante, Niamey, NER; 4 Public Health, Primary Health Care Development Board, Osogbo, NGA; 5 Anaesthesia, Osun State University, Osogbo, NGA; 6 Community Medicine, Institut Superieur de Sante, Niamey, NER

**Keywords:** awareness, maternal and child health, nigeria, osogbo, osun state, primary health centers, utilization

## Abstract

Background: Maternal and child health (MCH) services are crucial for the well-being of mothers and children, particularly in developing countries. However, awareness and utilization of these services can be impacted by various factors including socio-cultural beliefs, education, and access to healthcare. This study aims to examine the awareness and utilization of MCH services among women of reproductive age residing in Osogbo, Osun State.

Methods: A cross-sectional descriptive survey was conducted, involving 250 women of reproductive age selected through a multi-stage sampling technique. Data were collected using a structured questionnaire covering socio-demographic characteristics, awareness of MCH services, and utilization of these services. Both descriptive and inferential statistics were used to analyze the data.

Results: Awareness was high, with 232 (92.8%) participants aware of antenatal care (ANC) and 227 (90.8%) aware of child immunizations. Utilization rates were also high; 210 (84.0%) had ANC during their last pregnancy, and 207 (82.8%) immunized their last child. Major barriers included unavailability of vaccines (30, 12.0%), distance to the immunization site (54, 21.6%), lack of awareness of the need for immunization (11, 4.4%), fear of side reactions (7, 2.8%), absence of the vaccinator (2, 0.8%), postponement of the immunization (2, 0.8%), financial issues (4, 1.6%), inconvenient timing of immunization (7, 2.8%), lack of confidence in vaccines (1, 0.4%), and unfriendly vaccine providers (2, 0.8%). Utilization was significantly associated with age (OR: 2.34, p= 0.003, C.I. = 1.34 - 4.10), religion (OR = 0.33, *p* = 0.015, C.I. = 0.13 - 0.82), and awareness (OR: 3.99, p= 0.023, C.I. = 0.38 - 4.25).

Conclusion: While awareness and utilization of MCH services are generally high among women in Osogbo Metropolis, significant barriers still exist. There is a need for targeted interventions to address these barriers and improve access to MCH services. Enhancing education, improving service integration, and addressing logistical and financial obstacles are crucial for improving maternal and child health outcomes in this region.

## Introduction

The term maternal and child health (MCH) refers to the comprehensive means of providing supportive, preventive, curative, and rehabilitative health care to mothers and children. According to the World Health Organization (WHO), the elements of MCH include prenatal care, antenatal care (ANC), institutional births, qualified birth attendants, postpartum care, and child welfare/vaccination clinics [1]. Adequate access to and use of MCH services are critical to improving health outcomes and reducing maternal and child mortality rates [2].

Many countries have reduced their maternal mortality rates over the past three decades and contributed to the global decline in maternal mortality. From 1990 to 2017, maternal death rates decreased in Germany and France as well, with average yearly decreases of 3.4% and 3.7%, respectively. With average annual decreases of 3.8% between 1990 and 2017, Australia and Japan both continuously maintained low rates of maternal mortality [3]. However, in sub-Saharan Africa, where over 50% of all maternal deaths occur, maternal mortality rates have largely stagnated. Bleeding, abortion, sepsis, and obstructed labor are among the leading causes of maternal death in this region [4].

Nigeria, the most populous nation in Africa, presents significant obstacles in terms of mother and child health [5]. Despite various efforts by the government and international organizations to improve health care, the country continues to have high maternal and child mortality rates [6]. Factors such as limited access to quality healthcare facilities, inadequate infrastructure, poverty, cultural beliefs and practices, and low awareness of available services all contribute to this problem [5]. In Osogbo, Osun State, these issues are further compounded by uneven distribution of healthcare resources, urban-rural disparities, and limited targeted awareness campaigns.

Poor MCH outcomes extend beyond the health sector, creating significant societal consequences. Economically, the loss of mothers and children reduces workforce productivity, increases household poverty, and places a heavy burden on healthcare systems [2]. Educationally, maternal illness or death often disrupts children’s schooling, perpetuating cycles of illiteracy and poverty. At the community level, high maternal and child mortality undermines population growth stability, reduces the potential for human capital development, and strains public health resources; these effects are particularly visible in resource-limited urban centers like Osogbo [4].

However, there are gaps in the literature. While several national and regional studies have assessed MHC service utilization in Nigeria, few have explored the combined influence of awareness and utilization specifically within Osogbo, despite its unique socio-cultural and infrastructural context. Additionally, there is limited empirical evidence that directly links awareness levels to actual service uptake in this setting, making it difficult for policymakers to design targeted interventions. This study addresses these gaps by investigating both awareness and utilization patterns among women of reproductive age in Osogbo, with the aim of informing context-specific health improvement strategies.

## Materials and methods

This study employed a descriptive cross-sectional study design among women of reproductive age (15-49years) residing in Osogbo. Women of reproductive age (15-49 years) who have children and reside in Osogbo were included in the study, while women of reproductive age who do not have children and those who do not reside in Osogbo (visitors) and those unwilling to participate in the study were excluded. A sample size of 250 respondents was estimated using N = (pq)/ taken p as 83% from the study by Bassey and Sunday [7]. A multi-stage sampling technique was used to ensure representativeness across both local government areas (LGAs) in Osogbo while maintaining feasibility.

Stage 1: Two wards were purposively selected from each LGA to capture both central and peri-urban settings, which differ in service access and socio-economic characteristics.

Stage 2: From each ward, two major communities were randomly selected, giving a total of eight communities. This approach reduced geographical clustering bias and allowed inclusion of diverse demographic profiles.

Stage 3: Houses in each community were numbered, and those with odd numbers were selected systematically. This method minimized interviewer selection bias while maintaining randomness. Eligible respondents within selected households were then chosen by simple random sampling if more than one met the criteria.

While this method enhanced representativeness, systematic selection of odd-numbered houses could introduce bias if housing arrangements in some areas follow patterns (e.g., certain types of households are more likely to have odd addresses). To mitigate this, field teams verified that the numbering sequence was not systematically associated with socio-economic status.

Data were collected using a semi-structured, facilitated, self-administered questionnaire. The instrument measured the following constructs: (i) Sociodemographic characteristics - age, education, occupation, marital status, parity, and household income; (ii) Awareness of MCH services - knowledge of available services, service locations, and recommended schedules; (iii) Utilization patterns - frequency of antenatal, postnatal, immunization, and child welfare clinic attendance; (iv) Perceived barriers - financial constraints, distance, cultural beliefs, service quality perceptions; (v) Satisfaction with services - waiting times, staff attitude, availability of medicines and supplies.

The awareness index was constructed using three key indicators: knowledge of available MCH services, awareness of service locations, and understanding of recommended schedules such as antenatal visits and immunization timetables. Each correct or positive response was assigned one point, with a maximum score of three. Respondents who scored two to three points were categorized as having a high level of awareness, while scores of zero or one indicated low awareness.

The utilization index measured the actual uptake of MCH services based on four indicators: ANC attendance, postnatal care utilization, completion of age-appropriate immunizations, and participation in child welfare clinics. Each service attended in line with recommended guidelines scored one point, irregular or incomplete attendance scored 0.5, and non-attendance scored zero, giving a maximum possible score of four. Utilization was then categorized as good (scores 2-4, at least 50% of recommended services) or poor (scores <2, <50%).

Reliability and validity of the instrument

To ensure validity, questionnaire items were developed based on a review of existing literature and adapted from previously validated tools in similar settings. Content validity was established through expert review by two public health specialists and one maternal health researcher.

Reliability was assessed in two ways.

Internal Consistency

Cronbach’s alpha was calculated for key multi-item constructs, with values ≥0.70 considered acceptable.

Pre-testing

The questionnaire was pre-tested among 25 women (10% of the sample size) in Ede South LGA, a location with similar socio-demographic characteristics to Osogbo. Feedback from the pre-test informed revisions to improve clarity, cultural appropriateness, and response options.

Data were analyzed using IBM SPSS Statistics for Windows, Version 27 (Released 2020; IBM Corp., Armonk, New York, United States).

Univariate analysis (frequencies, percentages) summarized respondent characteristics and service utilization rates. Bivariate analysis using the chi-square test assessed associations between two categorical variables (e.g., education level and MCH service use). The chi-square test determines whether observed differences between groups are likely due to chance. Multivariate analysis using binary logistic regression identified independent predictors of MCH service utilization while controlling for potential confounders. Logistic regression is appropriate here because the main outcome (utilization) is binary (yes/no).

Statistical significance was set at p < 0.05, meaning that the probability of observing the result by chance is less than 5%.

Ethical approval to carry out the study was sought and obtained from the Health Research and Ethics Committee of the College of Health Sciences (CHS HREC), Osun State University. Participation of respondents in the study was completely voluntary. Written informed consent was obtained from each of the study respondents.

## Results

Sociodemographic characteristics

The age distribution reveals that the majority of the respondents, 140 (56.0%), were aged 20-29 years, while 101 (40.4%) were aged less than 20 years. In terms of religious affiliation, 123 (49.2%) identify as Christians, 109 (43.6%) identify as Muslims, while 18 (7.2%) identify as Traditional worshippers. The majority, 196 (78.4%), of the respondents are married, and 233 (93.2%) had less than five children. More than half of the respondents, 145 (58.0%), had tertiary education, 121 (48.4%) had spouses who had tertiary education and 88 (35.2%) are engaged in unskilled labor. Regarding proximity to healthcare facilities, 131 (52.4%) live within ≤ 5 km of a health facility, while 119 (47.6%) live > 5 km away (Table 1).

**Table 1:** Socio-demographic Characteristics Data has been presented as frequency and percentage

Variable (N=250)	Frequency	Percentage
Age		
< 20	101	40.4
20-29	140	56.0
30-49	9	3.6
Religion		
Christianity	123	49.2
Islam	109	43.6
Traditional	18	7.2
Ethnicity		
Yoruba	185	74.0
Hausa	31	12.4
Igbo	34	13.6
Marital status		
Single	26	10.4
Married	196	78.4
Separated	8	3.2
Divorced	14	5.6
Widowed	6	2.4
Educational Status		
No formal education	26	10.4
Primary	5	2.0
Secondary(SSCE)	74	29.6
Tertiary	145	58.0
Spouse Educational Status		
No formal education	42	16.8
Primary	11	4.4
Secondary (SSCE)	76	30.4
Tertiary	121	48.4
Spouse Job		
Unemployed	16	6.4
Unskilled Labour	88	35.2
Skilled Labour	76	30.4
Professional	70	28.0
Number of children		
<5	233	93.2
5-8	17	6.8
How far is the health facility		
≤ 5km	131	52.4
> 5km	119	47.6

Awareness of MCH services

The majority of the respondents, 232 (92.8%), were aware of the importance of ANC during pregnancy, 229 (91.6%) know where these services are available, 219 (87.6%) were informed about the recommended immunization schedule for children and 221 (88.4%) know where to access immunization services. Regarding awareness of other child health services, such as growth monitoring and nutritional counseling, 190 (76.0%) of the respondents were aware of these services. One hundred and sixty-three (65.2%) of the respondents primarily relied on healthcare providers for information on MCH (Table 2).

**Table 2:** Awareness of Maternal and Child Health Services Data has been presented as frequency and percentage MCH: Maternal and child health

Variable (N=250)	Frequency	Percentage
Are you aware of the importance of antenatal care during pregnancy		
Yes	232	92.8
No	18	7.2
Do you know where to access antenatal care services in your community?		
Yes	229	91.6
No	21	8.4
Aware of the importance of immunizations for children		
Yes	227	90.8
No	23	9.2
Do you know the recommended immunization schedule for children		
Yes	219	87.6
No	31	12.4
Do you know where to access immunization services for children in your community		
Yes	221	88.4
No	29	11.6
Are you aware of other child health services such as growth monitoring and nutritional counseling		
Yes	190	76.0
No	60	24.0
Source of information about MCH		
Healthcare Providers	163	65.2
Family and Friends	20	8.0
Internet	67	26.8

**Figure 1:**
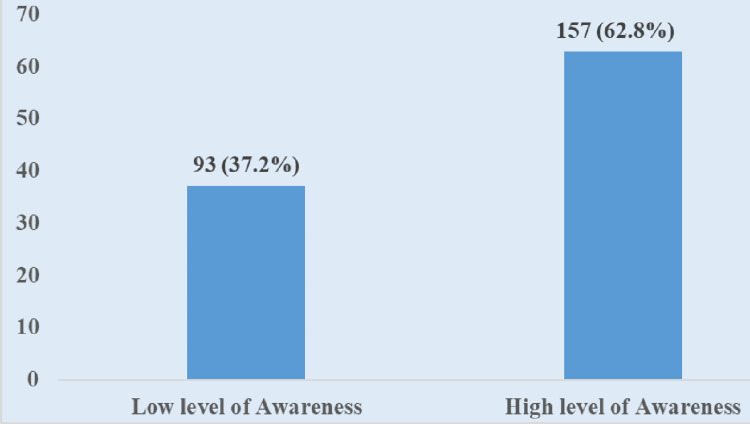
Overall Level of Awareness of Maternal and Child Health Services Among Respondents Data has been presented in frequencies and percentages

Overall, 157 (62.8%) of the respondents have a high level of awareness, while 93 (37.2%) have a low level of awareness regarding MCH services (Figure 1).

Utilization of MCH services

The majority of respondents, 210 (84.0%), attended antenatal clinics for their previous pregnancy out of which 113 (53.8%) started ANC within 4-6 months and 154 (61.6%) had five or more visits. Among those who did not attend antenatal clinics, reasons include financial constraints (9, 22.5%), distance to health facilities (11, 27.5%), staff attitudes (8, 20.0%), long waiting times (11, 27.5%), and unavailability of drugs (1, 2.5%).

More than half, 145 (58.0%), of the respondents delivered in public health facilities, while 24 (9.6%) used private health facilities. Forty-four respondents (17.6%) chose maternity centers, and others delivered with Traditional Birth Attendants, 18 (7.2%) or at home 19 (7.6%). Among those who did not deliver at health facilities, reasons include financial constraints (17, 45.9%) and distance to health facilities (14, 37.8%) and three (8.1%) had no ready means of transportation and the same proportion reported that it was their husband’s decision. The majority of the respondents, 208 (83.2%), went back to the health worker six weeks after delivery (Table 3).

**Table 3:** Utilization of Maternal Health Services Data has been presented as frequency and percentage ANC: Antenatal care; TBA: Traditional birth attendant

Variable (N=250)	Frequency	Percentage
Did you attend ANC during your last pregnancy?		
Yes	210	84.0
No	40	16.0
If no, why (n = 40)		
Cannot afford the cost of services	9	22.5
Health facilities are far	11	27.5
Poor attitude of Staff	8	20.0
Long waiting time	11	27.5
Drugs not available	1	2.5
At what month of the pregnancy did you start attending ANC (n = 210)		
1-3 months	88	41.9
4-6 months	113	53.8
7 months and above	9	4.3
How many ANC visits did you make before delivery (n = 210)		
≤ 5 visits	56	26.7
≥ 5 visits	154	73.3
Where did you deliver during your last pregnancy		
Public health facility	145	58.0
Private health facility	24	9.6
Maternity	44	17.6
TBA	18	7.2
Home	19	7.6
If you did not deliver in a health facility, why? (n=37)		
Could not afford	17	45.9
Health facilities too far	14	37.8
No ready means of transport	3	8.1
Husband’s decision	3	8.1
Did you go back to the health worker 6 weeks after delivery		
Yes	208	83.2
No	42	16.8
If yes, why (n = 208)		
Because I was ill	6	2.9
Because the baby needed its immunization	90	43.3
Because the midwife had told me I should	92	44.2
Because I wanted to start family planning	4	1.9
Because I wanted to make sure I am back to normal	16	7.7
If not, why? (n=42)		
Could not afford	12	28.6
Health facilities too far/ No ready means of transport	8	19.0
Husband’s decision	4	9.5
Don’t like the facility	18	42.9

The majority, 207 (82.8%), reported that their last child had been immunized. Among the immunized children, the vaccines received included BCG (204, 81.6%), OPV 0 (204, 81.6%), OPV 1 (185, 74.0%), OPV 2 (181, 72.4%), OPV 3 (174, 69.6%), PENTA 1 (166, 66.4%), PENTA 2 (164, 65.6%), PENTA 3 (170, 68.0%), Measles (152, 60.8%), Yellow Fever (123, 49.2%), and Vitamin A (132, 52.8%). For those children who did not complete their immunization schedule, the barriers included unavailability of vaccines (30, 12.0%), distance to the immunization site (54, 21.6%), lack of awareness of the need for immunization (11, 4.4%), fear of side reactions (7, 2.8%), absence of the vaccinator (2, 0.8%), postponement of the immunization (2, 0.8%), financial issues (4, 1.6%), inconvenient timing of immunization (7, 2.8%), lack of confidence in vaccines (1, 0.4%), and unfriendly vaccine providers (2, 0.8%) (Table 4).

**Table 4:** Utilization of Child Health Services Data has been presented as frequency and percentage. BCG: Bacillus Calmette-Guérin; OPV: Oral Polio Vaccine; PENTA: Pentavalent

Variable (N=250)	Frequency	Percentage
Was your last child ever immunized		
Yes	207	82.8
No	43	17.2
Which of these vaccines has he/she received		
BCG	204	81.6
OPV 0	204	81.6
OPV 1	185	74.0
OPV 2	181	72.4
OPV 3	174	69.6
PENTA 1	166	66.4
PENTA 2	164	65.6
PENTA 3	170	68.0
Measles	152	60.8
Yellow fever	123	49.2
Vitamin A	132	52.8
Why did your child not complete his/her immunization (n=137)		
Vaccine not available	30	12.0
Place of immunization too far	54	21.6
Unaware of the need for immunization	11	4.4
Fear of side reactions	7	2.8
Vaccinator absent	2	0.8
Postponed until next time	2	0.8
Lack of money	4	1.6
The time of immunization is not convenient	7	2.8
Lack of confidence and trust	1	0.4
Vaccine providers are not friendly	2	0.8
Long waiting times by mothers	19	7.6

Overall, 137 (54.8 %) of the respondents have good utilization, while 113 (45.2%) have poor utilization regarding MCH Services. 

Factors associated with utilization of MCH services

At the bivariate level, age (ꭓ2 = 6.192, df = 2, p = 0.013) and religion (ꭓ2 = 12.112, df = 2, p = 0.002) and awareness of MCH (ꭓ2 = 7.782, df = 1, p = 0.025) were statistically significant associated with utilization of MCH. At the multivariate level, age was significantly associated with utilization of MCH services such that respondents within age group 20-29 years were twice more likely to utilize MCH services than those less than 20 years (OR: 2.34, p= 0.003, C.I. = 1.34 - 4.10). Religion was also statistically significant with utilization of MCH services such that respondents who were Muslims (OR = 0.50, p = 0.005, 0.31 - 0.80) and Traditional worshippers (OR = 0.33, p = 0.015, C.I.= 0.13 - 0.82) were less likely to utilize MCH services than Christians. Awareness of MCH services was significantly associated with utilization of MCH services such that respondents who were aware of MCH services were four times more likely to utilize MCH services than those who were not (OR: 3.99, p= 0.023, C.I. = 1.38 - 4.25) (Table 5).

**Table 5:** Factors Associated with Utilization of Maternal and Child Health Services Using Multivariate Analysis R: Reference; *: significant

Variable	Odds Ratio	p-value	Confidence Interval
Age			
< 20 (R)	-	-	-
20-29	2.34	*0.003	1.34 – 4.10
30-49	1.65	0.064	0.97 – 2.80
Religion			
Christianity (R)	-	-	-
Islam	0.50	*0.005	0.31 – 0.80
Traditional	0.33	*0.015	0.13 – 0.82
Awareness of MCH services			
No (R)	-	-	-
Yes	3.99	*0.023	1.38 – 4.25

## Discussion

The age distribution in this study, with a majority of respondents aged 20-29years, reflects a demographic often described as being in their most active reproductive years. Similar to findings by Kebede et al. [8], this group’s increased awareness of MCH services is likely due to repeated pregnancies and sustained interaction with healthcare systems. However, unlike studies in Ethiopia that found younger mothers (<25 years) underutilized services mainly due to social stigma and lack of autonomy [9]. The findings of this study show that women aged 20-29 had higher utilization rates than adolescents, suggesting that stigma and decision-making power still disproportionately disadvantage the youngest mothers.

Religious affiliation did not strongly skew awareness levels, but its influence on service utilization was evident, with Muslims and traditionalists reporting lower uptake than Christians. This pattern aligns with the findings of Adedokun et al. [10], who observed that in parts of northern Nigeria, restrictive gender norms and preference for same-sex healthcare providers limit women’s access to skilled care. For traditionalists, lower utilization may stem from reliance on indigenous healing practices, mistrust of biomedical care, and the perception that childbirth is a natural process requiring minimal intervention. These cultural and religious dimensions underscore the importance of tailoring MCH outreach to respect belief systems while addressing misconceptions.

The high proportion of married respondents (78.4%) and their comparatively higher service utilization is consistent with Nuamah et al. [11], who reported that spousal support facilitates access to ANC and skilled delivery. The link between higher educational attainment-seen in over half of respondents and their spouses-and improved awareness of MCH services mirrors the conclusions of Tesema & Haimanot [12] and Agunwa et al. [13], suggesting that education equips women with the skills to navigate health systems effectively.

Proximity to health facilities emerged as a clear determinant of service uptake, consistent with Nuamah et al. [11] and Kim et al. [14]. Those living >5 km from facilities faced greater access challenges, echoing the Three Delays Model by Thaddeus & Maine [15], where delays in reaching care contribute to underutilization. The model also explains why financial constraints, long waiting times, poor staff attitudes, and unavailability of drugs-reported in this study-persist as barriers: they represent delays in seeking and receiving care.

Awareness gaps were particularly notable in growth monitoring and nutritional counseling. This finding is similar to the findings of Edae et al. [16] in Ethiopia, where health promotion focused heavily on ANC and immunization, leaving nutrition counseling underemphasized. Given that malnutrition remains a major contributor to under-five mortality in Nigeria, future health campaigns should rebalance messaging to highlight these services. Healthcare providers were identified as the primary source of MCH information by 163 (65.2%) of respondents. This is consistent with the findings by Adepoju et al. [17], who emphasized the central role of frontline health workers in improving maternal and child health outcomes through education during clinic visits.

Public health facilities were the preferred place of delivery for 58.0% of respondents, possibly due to perceived affordability, availability of trained staff, and relative accessibility. This contrasts with the findings of Estifanos et al. [18], who found that cost and perceived poor quality led many to seek care from private or informal providers. Similar findings have been documented in parts of Nigeria and Sub-Saharan Africa, where distrust in formal health systems, financial difficulties, and logistical challenges influence delivery choices [19]. For those choosing traditional birth attendants or home deliveries, our findings align with the findings of Nanur et al. [20], who identified trust, cultural familiarity, and flexible payment arrangements as key drivers.

The majority of respondents (210, 84.0%) attended antenatal clinics during their last term of pregnancy, with over half initiating visits between the fourth and sixth months of gestation and most having five or more visits. These findings align with World Health Organization (WHO) recommendations advocating a minimum of four ANC visits to ensure optimal maternal and fetal health outcomes [1]. However, late booking remains a concern and may hinder early detection and management of pregnancy complications. Similar trends have been reported in studies conducted in Nigeria and other low- and middle-income countries where cultural beliefs, limited knowledge, and systemic barriers delay ANC initiation [18,21].

PNC and immunization uptake were also relatively high, yet drop-off for later childhood vaccines was observed. This is consistent with previous research in Nigeria, which found that while BCG and early OPV doses are widely accepted, completion of the full schedule remains suboptimal due to factors such as vaccine stock-outs and travel distance [10,22]. This suggests that while initial contact with MCH services is strong, continuity is undermined by systemic inefficiencies.

From a theoretical perspective, the Health Belief Model (HBM) helps explain why these barriers persist. Perceived susceptibility (e.g., not recognizing risks of unskilled birth), perceived severity (e.g., underestimating complications from delayed ANC), perceived benefits (e.g., viewing traditional care as equally effective), and perceived barriers (e.g., cost, distance, waiting times) all interact to influence health-seeking behavior. Addressing these requires interventions that enhance risk perception, demonstrate tangible benefits of formal care, and reduce logistical and financial obstacles.

In this study, 137 (54.8%) of respondents had good utilization of MCH services, indicating room for improvement. Statistically significant predictors of MCH service utilization included age, religion, and awareness of services. Women aged 20-29 were more likely to utilize MCH services than those under 20, consistent with findings that adolescent mothers often face additional stigma, lack of autonomy, and limited access to reproductive health information [23]. Religious affiliation also influenced utilization, with Muslims and traditionalists being less likely to access MCH services compared to Christians. This may reflect variations in cultural norms, gender dynamics, and trust in biomedical healthcare, as documented in prior studies from northern Nigeria [4,9].

Importantly, awareness emerged as a strong positive predictor of utilization: women who were aware of MCH services were nearly four times more likely to use them. This underscores the critical role of health education and community outreach in improving uptake of services [23]. Tailored health promotion programs that consider local contexts, literacy levels, and belief systems are essential to increasing service utilization.

Future research directions

Cultural and Religious Influences

More in-depth qualitative studies are needed to explore how belief systems among traditionalists and certain religious groups influence attitudes toward formal MCH services.

Preference for Public Hospitals

Investigating whether preferences are driven by cost, trust in government systems, or proximity would help refine service delivery strategies.

Continuity of Care

Understanding why immunization completion drops despite high initial uptake could guide supply chain and outreach improvements.

Targeted Awareness Campaigns

Research should evaluate the impact of expanding health education to underrepresented services such as nutrition counseling and growth monitoring.

Interventions for Adolescents

Given their lower utilization, targeted adolescent-friendly MCH services should be assessed for effectiveness in improving early ANC attendance and skilled delivery.

Strengths and limitations of the study

A key strength of this study is its use of a multi-stage sampling technique that enhanced representation across both central and peri-urban areas of Osogbo, capturing diverse socio-economic and cultural contexts. The study also benefited from a relatively large sample size (250 respondents), improving the reliability of its estimates. Additionally, the use of a validated, pre-tested questionnaire with acceptable internal consistency (Cronbach’s alpha ≥ 0.70) ensured data quality and cultural appropriateness. The combination of descriptive, bivariate, and multivariate analyses allowed for a comprehensive examination of factors associated with MCH service utilization, providing actionable insights for policymakers and health planners.

This study has some limitations that should be considered when interpreting the findings. The cross-sectional design limits the ability to establish causal relationships between awareness and utilization of MCH services. Associations observed may be influenced by unmeasured confounders such as prior health conditions, household decision-making dynamics, or exposure to previous health campaigns. The purposive selection of wards and systematic sampling of odd-numbered houses may have introduced selection bias, potentially limiting representativeness. Restricting participants to women of reproductive age who already had children excluded nulliparous women and adolescents, whose experiences may differ. Self-reported data are subject to recall bias, particularly for events that occurred in the past, and social desirability bias may have led to over-reporting of positive health behaviors such as ANC attendance or child immunization. Finally, the findings are context-specific to socio-cultural and infrastructural setting in Osogbo and may not be generalizable to other regions with different characteristics. 

Future research should adopt longitudinal designs to track how awareness influences service utilization over time, include a broader range of women such as nulliparous and adolescent females to capture diverse experiences, and use mixed-methods approaches to explore cultural and personal factors affecting MCH service uptake. Studies should also validate self-reported data with health facility records to minimize recall and social desirability bias, ensuring more accurate and reliable findings.

Actionable recommendations for stakeholders

Government Agencies

Government agencies should strengthen the supply chain for vaccines and essential drugs to prevent stock-outs and expand community-based MCH service delivery points to reduce the travel burden for women living far from facilities.

Primary Health Care Development Boards

Primary Health Care Development Boards should implement regular health education campaigns, particularly targeting communities with lower educational attainment and minority religious groups where uptake is lower.

Non-Governmental Organizations (NGOs)

NGOs should partner with local religious and community leaders to address cultural barriers and build trust in formal health services.

Healthcare Providers

Healthcare providers should Improve staff attitudes and reduce waiting times through continuous professional development and better patient flow management.

## Conclusions

The high awareness and relatively good utilization of MCH services observed in this study are encouraging but should not obscure the persistent gaps - especially in timely ANC initiation, complete immunization coverage, and equitable access for all socio-economic and religious groups. Addressing these issues requires urgent, coordinated action from policymakers, healthcare providers, and community stakeholders. Without targeted interventions to overcome the identified barriers, progress toward reducing maternal and child morbidity and mortality will remain slow. There is a need to invest in sustainable, culturally sensitive, and accessible MCH programs to improve health outcomes for mothers and children in Osogbo and similar settings.

## Appendices

Questionnaire

Dear Respondent, This study is on ‘Awareness and Utilization of Maternal and Child Health Services among Women of Reproductive Age Residing in Osogbo, Osun State’. The information requested in this questionnaire is strictly for academic purposes. Absolute anonymity and confidentiality are guaranteed. Please read the questions carefully and answer them with all honesty, as your sincere response will be of great help to the success of the study. Thank you.

Kindly sign here if you agree to take part in this study ____________________

Section A: Socio-demographic Characteristics

1. Age (as at last birthday) ______________

2. Religion (A) Christianity (B) Islam (C) Traditional

3. Ethnicity (A) Yoruba (B) Hausa (C) Igbo (D) Others

4. Marital Status (A) Single (B) Married (C) Separated (D) Divorced (E) Widowed

5. Educational Status (A) No formal education (B) Primary (C) Secondary (SSCE) (D) Tertiary

6. Spouse’s Educational Status (A) No formal education (B) Primary (C) Secondary (SSCE) (D) Tertiary

7. Spouse’s Occupation (A) Unemployed (B) Unskilled labour (C) Skilled labour (D) Professional

8. Number of children __________

9. Distance to the nearest health facility: (A) ≤ 5 km (B) > 5 km

Section B: Awareness of Maternal and Child Health Services

10. Are you aware of the importance of antenatal care during pregnancy? (A) Yes (B) No

11. Do you know where to access antenatal care services in your community? (A) Yes (B) No

12. Are you aware of the importance of immunizations for children? (A) Yes (B) No

13. Do you know the recommended immunization schedule for children? (A) Yes (B) No

14. Do you know where to access immunization services for children in your community? (A) Yes (B) No

15. Are you aware of other child health services such as growth monitoring and nutritional counseling? (A) Yes (B) No

16. What is your source of information about MCH services? (Tick all that apply) (A) Healthcare providers (B) Family and friends (C) Internet

Section C: Utilization of Maternal and Child Health Services

17. Did you attend ANC during your last pregnancy? (A) Yes (B) No

18. If No, why not? (A) Cannot afford (B) Facility far (C) Poor staff attitude (D) Long waiting time (E) Drugs not available

19. At what month of the pregnancy did you start attending ANC? (A) 1-3 months (B) 4-6 months (C) 7 months and above

20. How many ANC visits did you make before delivery? (A) < 5visits (B) ≥ 5 visits

21. Where did you deliver your last child? (A) Public facility (B) Private facility (C) Maternity (D) TBA (E) Home

22. If not in a health facility, why? (A) Could not afford (B) Facility too far (C) No transport (D) Husband’s decision

23. Did you go back to a health worker 6 weeks after delivery? (A) Yes (B) No

24. If Yes, why? (A) Illness (B) Baby’s immunization (C) Midwife instruction (D) Start family planning (E) Check health status

25. If No, why not? (A) Could not afford (B) Facility too far/No transport (C) Husband’s decision (D) Don’t like the facility

Section D: Child Immunization

26. Was your last child ever immunized? (A) Yes (B) No

27. Which vaccines has your child received? (Tick all that apply) (A) BCG (B) OPV 0 (C) OPV 1 (D) OPV 2 (E) OPV 3 (F) PENTA 1 (G) PENTA 2 (H) PENTA 3 (I) Measles (J) Yellow fever (K) Vitamin A

28. If your child did not complete immunization, why? (Tick all that apply) (A) Vaccine not available (B) Facility too far (C) Unaware (D) Fear of side effects (E) Vaccinator absent (F) Postponed (G) Lack of money (H) Time not convenient (I) No confidence/trust (J) Unfriendly staff (K) Long waiting times
